# CD74 regulates complexity of tumor cell HLA class II peptidome in brain metastasis and is a positive prognostic marker for patient survival

**DOI:** 10.1186/s40478-018-0521-5

**Published:** 2018-03-01

**Authors:** P. S. Zeiner, J. Zinke, D. J. Kowalewski, S. Bernatz, J. Tichy, M. W. Ronellenfitsch, F. Thorsen, A. Berger, M. T. Forster, A. Muller, J. P. Steinbach, R. Beschorner, J. Wischhusen, H. M. Kvasnicka, K. H. Plate, S. Stefanović, B. Weide, M. Mittelbronn, P. N. Harter

**Affiliations:** 1 Edinger Institute (Institute of Neurology), Goethe-University, Heinrich-Hoffmann-Str. 7, D-60528 Frankfurt am Main, Germany; 2 Dr. Senckenberg Institute of Neurooncology, Goethe-University, Frankfurt am Main, Germany; 3 Department of Immunology, Institute for Cell Biology, University of Tuebingen, Tuebingen, Germany; 4 Immatics Biotechnologies GmbH, Tübingen, Germany; 5 Department of Biomedicine, The Kristian Gerhard Jebsen Brain Tumour Research Center and The Molecular Imaging Center, University of Bergen, Bergen, Norway; 6 Institute for Virology, Goethe-University, Frankfurt am Main, Germany; 7 Department of Neurosurgery, Goethe-University, Frankfurt am Main, Germany; 8 Department of Oncology, Luxembourg Institute of Health, Luxembourg, Luxembourg; 9 German Cancer Research Center DKFZ Heidelberg, Germany and German Cancer Consortium DKTK partner site, Frankfurt/Mainz, Germany; 10 Department of Pathology and Neuropathology, University of Tuebingen, Tuebingen, Germany; 11 Department of Gynecology, University of Wuerzburg, Wuerzburg, Germany; 12 Goethe-University, Dr. Senckenberg Institute for Pathology, Frankfurt am Main, Germany; 13 Department of Dermatology, University of Tuebingen, Tuebingen, Germany; 14 Luxembourg Centre of Neuropathology (LCNP), 3555 Dudelange, Luxembourg; 15 Laboratoire National de Santé, Department of Pathology, 3555 Dudelange, Luxembourg; 16 Luxembourg Centre for Systems Biomedicine (LCSB), University of Luxembourg, 4361 Esch-sur-Alzette, Luxembourg; 17 NORLUX Neuro-Oncology Laboratory, Department of Oncology, Luxembourg Institute of Health (L.I.H.), 1526 Luxembourg, Luxembourg

**Keywords:** CD74, HLA class II, Brain metastasis, HLA peptidome, Tumor infiltrating lymphocytes

## Abstract

Despite multidisciplinary local and systemic therapeutic approaches, the prognosis for most patients with brain metastases is still dismal. The role of adaptive and innate anti-tumor response including the Human Leukocyte Antigen (HLA) machinery of antigen presentation is still unclear. We present data on the HLA class II-chaperone molecule CD74 in brain metastases and its impact on the HLA peptidome complexity.

We analyzed CD74 and HLA class II expression on tumor cells in a subset of 236 human brain metastases, primary tumors and peripheral metastases of different entities in association with clinical data including overall survival. Additionally, we assessed whole DNA methylome profiles including CD74 promoter methylation and differential methylation in 21 brain metastases. We analyzed the effects of a siRNA mediated CD74 knockdown on HLA-expression and HLA peptidome composition in a brain metastatic melanoma cell line.

We observed that CD74 expression on tumor cells is a strong positive prognostic marker in brain metastasis patients and positively associated with tumor-infiltrating T-lymphocytes (TILs). Whole DNA methylome analysis suggested that CD74 tumor cell expression might be regulated epigenetically via CD74 promoter methylation. CD74^high^ and TIL^high^ tumors displayed a differential DNA methylation pattern with highest enrichment scores for antigen processing and presentation. Furthermore, CD74 knockdown in vitro lead to a reduction of HLA class II peptidome complexity, while HLA class I peptidome remained unaffected.

In summary, our results demonstrate that a functional HLA class II processing machinery in brain metastatic tumor cells, reflected by a high expression of CD74 and a complex tumor cell HLA peptidome, seems to be crucial for better patient prognosis.

**Electronic supplementary material:**

The online version of this article (10.1186/s40478-018-0521-5) contains supplementary material, which is available to authorized users.

## Introduction

Brain metastases (BM) are the most frequent brain tumors in humans. Despite multimodal therapies including radio-chemotherapy, neurosurgery and/or stereotactic irradiation patient survival is still poor, often not exceeding 6–12 months [3, 43]. During the last years clinical trials focusing on modulation of the immune response (mostly by targeting immune checkpoints) have shown promising results in peripheral tumors of different cancer entities [13, 37, 55]. Unfortunately, knowledge about treatment response in BM is still poor. Recently, Frenard and colleagues showed that ipilimumab treatment (CTLA-4-dependent checkpoint-inhibitor) failed to prevent metastases formation in the per se immune privileged environment of the brain in patients suffering from metastatic melanoma [12] despite a potentially enhanced systemic immune response. Nevertheless, it has recently been shown that the PD-1 antibodies nivolumab and pembrolizumab might have significant activity in BM patients, indicating a potential tumor control function in BM of melanoma patients [34]. Interestingly, it has been described that the mutational load of metastatic melanomas predicts a better response to CTLA-4 blockade [41]. Likewise, hypermutated tumors with DNA mismatch-repair gene defects respond significantly better to PD-1 blockade as compared to tumors without DNA mismatch-repair gene defects and lower mutational load [25]. Even across different tumor entities, the response to immunotherapy is associated with mutational load as presented in humans via human leukocyte antigen (HLA) molecules [2]. This indicates that the mutational landscape presented via HLA molecules might be crucial for an adequate immune and thus therapy response.

Antigens are presented either via HLA class I or class II molecules. Tumor cell-derived (neo)-antigens are presented by the ubiquitously expressed HLA class I molecules, although recent data demonstrates murine mutant epitopes also on major histocompatibility complex (MHC) class II molecules [22]. HLA class II presentation is usually found on antigen presenting cells such as dendritic cells, macrophages and microglial cells. The expression of HLA class II molecules is not exclusively restricted to immune cells, HLA class II molecules have been described on cancer cells already several decades ago [8, 24, 48] and the loss of heterozygosity in HLA does correlate with tumor immune escape [29]. Further, HLA class II-dependent help from CD4-positive TILs might significantly support anti-tumor immune response [1] (the term TILs will be used throughout the manuscript describing the population of tumor infiltrating T-lymphocytes mainly consisting of CD3-, CD4- and CD8-positive cells). However, the clinical relevance of HLA class II expression on cancer cells might depend on the cancer type. Whereas e.g. in colorectal cancer HLA class II expression has been described to be associated with a favorable clinical outcome [23, 31, 40], HLA class II expression on e.g. melanomas or sarcomas has been linked with poor clinical outcome [30, 46]. Still there is the question whether the mere amount of HLA class II molecules on cancer cells is mediating these effects or weather modulations of the antigen processing and assembly machinery are resulting in differential presentation of immunogenic antigens on the cell surface.

CD74, also known as invariant chain (li) is essential for assembly and stabilization during HLA class II antigen presentation. HLA class II molecules are synthesized in the endoplasmic reticulum (ER) where they assemble with CD74, the latter being involved in proper folding and stabilization of HLA class II molecules. CD74 also facilitates the export of HLA class II complexes from the ER to lysosomal compartments where antigens are loaded and CD74 becomes proteolytically degraded by cathepsins leaving the CLIP peptide in the HLA class II binding groove. Although CD74 has recently been shown to be involved in cross-presentation on HLA class I molecules, the main function seem to be the chaperoning during HLA class II antigen presentation [4, 52]. CD74 itself is upregulated in a variety of peripheral (e.g. melanoma: [47], lung cancer: [28]) and especially hematopoietic neoplasms [6, 7]. Several studies in peripheral tumors suggest proneoplastic effects of CD74 [9, 20, 27] or even show a negative association of CD74 expression with overall survival in vivo [33]. On the contrary other studies observed controversial CD74 effects with prolonged overall survival and an association with HLA class II expression [11, 54]. The exact mechanisms how CD74 might functionally influence neoplastic behavior remain multifaceted including the function as a receptor for macrophage migration inhibitory factor (MIF) [26] and a predominant expression on non-neoplastic antigen-presenting cells of the microenvironment in some tumor entities [26].

Here we analyze expression profiles and clinical relevance of the major assembly and stabilization molecule CD74 in a large cohort of BM patients. Furthermore, we analyzed the consequences of CD74 expression on the HLA peptidome of a brain metastatic cancer cell line.

## Material and methods

### Patient tissue and tissue microarrays (TMAs)

Data analyses was performed on paraffin embedded tissue samples which were processed to tissue micro arrays (TMAs) (deriving from the UCT tumor bank, Goethe-University, Frankfurt am Main, Germany, member of the German Cancer Consortium (DKTK), Heidelberg, Germany and German Cancer Research Center (DKFZ), Heidelberg, Germany or from the local biobank “Blut-und Gewebebank zur Erforschung des malignen Melanoms” Department of Dermatology, University Hospital Tuebingen, Tuebingen, Germany). Written consent was obtained from each patient. The study protocol was endorsed by the local ethical committee (Goethe-University Medical School/UCT Frankfurt GS-04/09, SNO-08-2015). In total, we investigated 236 embedded tissue samples including BM of: melanoma (*n* = 96), non small cell lung cancer (NSCLC, *n* = 56), breast carcinoma (*n* = 31), renal cell carcinoma (RCC, *n* = 18), small cell lung cancer (SCLC *n* = 8), colon carcinoma (*n* = 10), carcinomas which were not otherwise specified (carcinoma NOS *n* = 8) and specimens of rare tumors summarized as others (*n* = 9). Histopathology of BM was performed by board certified neuropathologists (KHP, MM and PNH). Histopathology of primary tumors and peripheral metastases was performed by a board certified pathologist (HMK). Further clinical data is shown in Additional file 1: Figure S1.

### Immunohistochemistry (IHC) and immunofluorescence (IF)

For immunohistochemical and immunofluorescent analyses of TMAs, whole mount sections and paraffin embedded cell pellets (of melanoma cell lines H1_DL2 (human brain seeking cell line deriving from melanoma BM) [45, 53], SK-MEL-2 (human skin metastatic melanoma), SK-MEL-28, UACC-257 (both human melanoma), the breast cancer cell lines MDA-MB-231 and JIMT-1 (both brain seeking clones of human pleural metastatic breast cancer) and the lung adenocarcinoma cell line PC14-PE6 (brain seeking clone of human lung adenocarcinoma)) the following antibodies were used: anti-CD3 (Dako A0452, dilution for IHC 1:500), anti-CD4 (Roche #790–4423, clone SP35, undiluted), anti-CD8 (Dako, clone C8/144B, dilution for IHC 1:100), anti-CD74 (Abcam, ab9514, dilution for IHC/IF 1:100/1:200), anti-Ki67 (Dako M7240, dilution for IHC 1:200) anti-HLA class II (Dako M0775, dilution for IHC/IF 1:1000 detecting HLA-DP, DQ, and DR) and anti-panCK (Dako, clone MNF116, dilution for IHC/IF 1:1000). TMA tissue blocks were cut in slices of 3 μm thickness using a microtome (Leica Microsystems, Nussloch GmbH, Nussloch, Germany) and placed onto SuperFrost slides (Thermo Scientific, Dreieich, Germany). IHC was performed according to standardized protocols using the Discovery XT automated immunostaining system (Ventana Medical Systems, München, Germany). IHC stainings were analyzed using a light microscope (BX41, Olympus, Hamburg, Germany). IF stainings were performed as described previously [57] and were evaluated using the Eclipse 80i fluorescent microscope (Nikon Eclipse 80i, Nikon, Japan).

### Statistical analyses of tissue micro array data

Quantification of CD4-positive TILs was performed on all BM with regard to positive lymphocytic cells related to all cells, while the amount of CD3-, CD8- and PD-1-positive TILs has already been calculated and described [16]. CD74- and HLA class II- (both intracellular and membrane-associated) positive tumor cells related to the total cell number were calculated using a semi quantitative IHC H-score (“histo” score) ranging from 0 to 300. Each staining intensity level (1 = weak, 2 = moderate, 3 = strong) and the percentage of positively stained cells in these particular levels (1, 2 or 3) were determined in the whole tissue sample. The staining intensity levels were then multiplied with the frequency of positively stained cells (in %). Finally, these scores per level were put together, ending up with a final score ranging from 0 to 300. PD-L1 expression on tumor cells has already been described [16]. CD74 expression in tumor cells was compared to clinical parameters such as, overall survival, Karnofsky Performance Status (KPS) or in case of melanoma the Graded Prognostic Assessment (GPA) score. If not otherwise stated, *p*-values are indicated including their 95% confidence intervals (*p < 0.05; **p < 0.01; ***p < 0.0001). A significance level of alpha =0.05 was selected. Statistical analyses were performed using JMP 11.0 software (SAS, Cary, NC, USA). Graphics were prepared using GraphPad Prism 6 software (GraphPad Software, Inc., La Jolla, CA, USA).

### CD74 siRNA knockdown

The melanoma brain metastasis cell line H1 which shows a tropism for the brain was grown in DMEM GlutaMax (Invitrogen) supplemented with 10% Fetal Bovine Serum (FBS Superior, Biochrome) and 1% Penicillin-Streptomycin (P/S, Sigma-Aldrich) at 37 °C and 5% CO2. Cells were seeded directly into the transfection mix consisting of DMEM (without FBS and P/S) and siRNA pools against human CD74 (NCBI gene ID: 972, sp972_5) in a final concentration of 6 nM for 96 h according to the manufacturer’s protocol. Unspecific (control siPools) served as a control condition (siTOOLs Biotech GmbH, Munich, Germany) [15]. LipofectamineTM 2000 (Invitrogen, Darmstadt, Germany) was used as a transfection reagent (5 μL in a six-well format, 30 μL in a 10-cm-format or T-175 flasks). To generate cyto pellets 2 × 10^6^ cells were seeded in a 10 cm petri dish applying a total volume of 10 ml/dish including transfection reagent, siRNA pools and DMEM. For extraction of RNA for qRT-PCR and RNA microarray as well as protein for immunoblotting 3 × 10^5^ cells/well of a 6-well plate in a total volume of 2 ml/well were seeded. These experiments were performed in triplicates. For peptidome analysis 8 × 10^6^ cells were seeded in T-175 cell culture flasks in a final volume of 15 ml (11xT-175 flasks per condition: siRNA pools against human CD74 versus unspecific control siPools, including each an additional flask for validation with immunoblotting as well as qRT-PCR).

### Quantitative-RealTime-PCR (qRT-PCR)

Total RNA was extracted according to the manufacturer’s protocol of the RNeasy Mini Kit (Qiagen, Hilden, Germany) from several metastatic cancer cell lines (melanoma cell lines: H1_DL2, SK-MEL-2, SK-MEL-28, UACC-257, breast cancer cell lines: MDA-MB-231, Jimt-1 and the lung adenocarcinoma cell line PC14-PE6) as well as the H1 cell line after CD74 knockdown with siRNA pools. The concentration of total RNA was determined photometrically with the NanoDrop™ 2000 spectral photometer (Thermo Scientific, Dreieich, Germany). Reverse transcription of 1 μg of RNA into complementary DNA (cDNA) was performed according to the manufacturer’s protocol of the RevertAidTM H Minus First Strand cDNA synthesis Kit (Thermo Scientific, Dreieich, Germany) using random hexamer primers. To digest template RNA after cDNA synthesis 1 μl of Ribonuclease H was added and incubated for 30 min at 37 °C. The quantitative polymerase chain reaction (qPCR) reactions were prepared in a final volume of 20 μ using SYBR green master mix (Thermo Fisher Scientific, Waltham, MA, USA) on a MyiQ Single Color Real-Time PCR Detection System (BIO-RAD, Hercules, CA, USA). CD74_551 (fw 5’-CCCGGAGAACCTGAGACACCT-3′, rv 5’-CCAAGGAGTGCCTGCTCATT-3′) and the internal standard control RPLP0 (fw 5’-GAGTCCTGGCCTTGTCTGTGG-3′, rv 5’-TCCGACTCTTCCTTGGCTTCA-3′) were designed as described previously [57]. Analyses were performed in triplicates. ΔCT and ΔΔCT values were determined.

### TaqMan® Array human antigen processing and presentation by MHCS

On the gene signature plate TaqMan® Array Human Antigen Processing and Presentation by MHCS (Fisher Scientific, Schwerte, Germany) 44 genes related to antigen processing and presentation as well as 4 endogenous control genes were tested in duplicates per condition according to the manufacturer’s protocol using the TaqMan® Gene Expression Master Mix (Fisher Scientific, Schwerte, Germany) on a MyiQ Single Color Real-Time PCR Detection System (BIO-RAD, Hercules, CA, USA). The aforementioned array was performed in the brain seeking melanoma metastasis cell line H1 after CD74 knockdown with siRNA pools. Unspecific control siPools (negative pools) served as a control condition. ΔCT and ΔΔCT values were determined.

### RNA microarray

RNA microarray analyses were performed using the brain seeking melanoma brain metastasis cell line H1 after CD74 knockdown with siRNA pools (see above). Unspecific control siPools (negative pools) served as control. Analyses were performed in triplicates. The comprehensive RNA microarray using the HumanHT-12 v4 Expression BeadChip Kit was completed at the Genomics and Proteomics Core Facility at the German Cancer Research Center, Heidelberg, Germany (DKFZ). Major transcripts of HLA class II components were analyzed for confirmation of TaqMan® data.

### Immunoblot analysis

Protein lysates of H1 cells after CD74 knockdown with siRNA pools were generated as described previously [18]. Unspecific control siPools (neg. pools) served as a control condition. Protein concentration was determined by using the Pierce® BCA Protein Assay Kit (Thermo Scientific, Dreieich, Germany) according to the manufacturer’s instructions. Electrophoretic separation of denatured proteins was performed on 15% SDS-polyacrylamide-gels using the Bio-Rad (Bio-Rad, München, Germany) electrophoresis system, followed by immunoblotting and immunodetection as described previously [57]. The following antibodies were used: anti-CD74 (Abcam, ab9514, dilution for WB 1:50), as a loading control anti-Lamin B1 (Abcam, ab16048, dilution for WB 1:4500). Immunoblots were developed with the Odyssey Fc (LI-COR, Lincoln, NE, USA). For quantitative analysis of immunoblots a densitometry approach was used as previously described with normalization of CD74 signal to Lamin B1 signal [18].

### Flow cytometry (FACS)

Membranous CD74 (anti-CD74, abcam, ab9514, dilution for FACS 1:50) expression of the brain seeking melanoma metastasis cell line H1 and the melanoma cell line SKMEL-28 was tested by FACS (FACSCanto-II flow cytometer (BD Bioscience)) against the positive control Raji as described previously [57]. HLA class II (anti-HLA-DR, Biolegend, clone L243, dilution for FACS 1:100) cell surface expression was assessed in H1 cells after CD74 knockdown with siRNA pools, unspecific control siPools (negative pools) serving as control treatment condition. An anti-mouse IgG1 antibody (Dako, Hamburg, Germany) was used as an isotype control for CD74 stainings and an anti-mouse IgG2a antibody (Dako, Hamburg, Germany) for HLA class II stainings respectively. Data were analyzed by Flow Jo software (TreeStar, Ashland, OR, USA).

### Whole DNA methylome analyses and CD74 promoter methylation status

CD74 promoter associated CpGs were analyzed in 21 NSCLC BM using the EPIC 850 k whole methylome Chip (Illumina, San Diego, USA) following standard protocols for tissue and DNA processing. Hybridization was performed as indicated by the manufacturer. Data were preprocessed using Illumina Genome Studio, further analysis was performed using JMP 11.0 (SAS, Cary, NC, USA). Mean beta-values were compared between CD74 high (*n* = 11) and low (*n* = 10) expressors as assessed in IHC (median H-Score 20). The following target promoter associated CpG sites were analyzed: cg01601628, cg11619961, cg11915469, cg13362637, cg14484145, cg16591228, cg18664712, cg19928046, cg19966212, cg22975568, cg24548564, cg26129545. Whole methylome analysis was performed using Partek Genomic Suite software (Partek Incorborated, St. Louis, Missouri, USA). We hypothetically stratified both CD74^high^ and TIL^high^ tumors versus tumors not showing these features. Differential methylation was performed using M-values after functional normalization. CpGs were regarded as differentially methylated with an unadjusted *p*-value of *p* < 0.0001. Differentially methylated CpGs were further processed with Partek Gene Ontology (GO) enrichment analysis.

### HLA peptidome analysis by mass spectrometry

HLA class I and class II molecules were isolated from 5 × 10^7^ CD74siRNA treated and mock treated H1 cells using standard immunoaffinity purification as described previously [21]. We independently transfected nine replicates each of control and siRNA knockdown condition (nine T175 flasks each). Due to the large amounts of cells which are necessary for sufficiently deep HLA peptidome analysis, we pooled all cells for each condition. Mass spectrometry was performed in 5 technical replicates for each condition to allow for statistical evaluation of label-free quantitation data. The pan-HLA class I-specific mAb W6/32 was utilized for isolation of class I molecules. A 1:1 mixture of the pan-HLA class II-specific mAb Tü-39 and the HLA-DR-specific mAB L243 was utilized for the isolation of HLA class II.

Label-free relative quantitation (LFQ) of the HLA peptidome composition was performed by LC-MS analysis of HLA class II ligand extracts from treated and control cells in five technical replicates. Peptide samples were separated by nanoflow high-performance liquid chromatography (RSLCnano, Thermo Fisher Scientific) using a 50 μm × 25 cm PepMap C18 column (Thermo Fisher Scientific) and a linear gradient ranging from 2.4% to 32.0% acetonitrile over the course of 90 min. Eluting peptides were analyzed in an online-coupled Orbitrap Fusion Lumos mass spectrometer (Thermo Fisher Scientific) in data dependent acquisition mode using collision-induced dissociation fragmentation. MS2 spectra for 2+ and 3+ precursors of 400–650 m/z were acquired at 30 k resolution with AGC target values of 70,000 and maximum injection times of 150 ms. Normalized collision energy was set to 35%, dynamic exclusion was set to 7 s. On column peptide amounts for the 5 LFQ replicates were adjusted based on initial dose-finding LC-MS analysis.

Database search was performed using SequestHT node of ProteomeDiscoverer 1.4.1.14 (Thermo Fisher Scientific) to search against the human proteome as comprised in the Swiss-Prot database (version of September 27th 2013, 20,279 reviewed protein sequences contained) without enzymatic restriction. Precursor mass tolerance was set to 5 ppm, fragment mass tolerance to 0.02 Da. Oxidized methionine was allowed as a dynamic modification. The false discovery rate (FDR) was estimated using Percolator [19] and limited to 1%. Peptide lengths were to 8–25 amino acids.

Relative quantification of HLA ligands was performed based on the peak area of the corresponding precursor MS1 extracted ion chromatogram. Imputation of missing values, 2-tier normalization for technical variability in MS signal intensity and calculation of fold-change and *p*-values (heteroskedastic t-test using Benjamini-Hochberg correction) for differential peptide presentation was performed using an in-house R script.

The mass spectrometry proteomics data have been deposited to the ProteomeXchange Consortium via the PRIDE [51] partner repository with the dataset identifier PXD008937.

## Results

### CD74 is expressed by tumor cells in BM and is associated with a better overall survival in vivo.

**Fig. 1:**
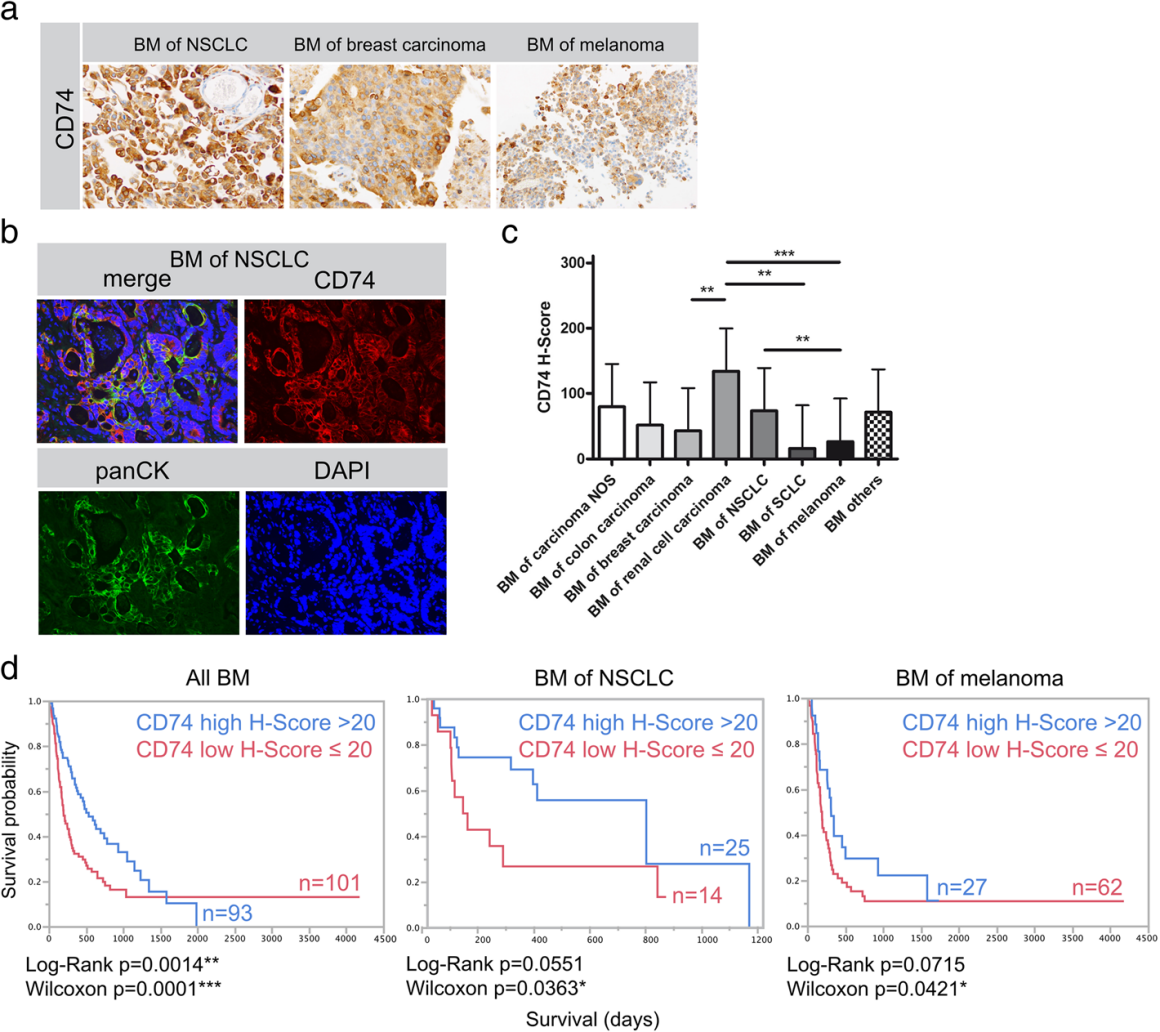
CD74 expression in human brain metastases. **a** Immunohistochemical staining against CD74 in three examples of BM from NSCLC, breast carcinoma and melanoma. **b** Example for CD74 and panCK showing co-expression in a NSCLC BM. **c** H-Score mean values and standard error of the mean (SEM) of all investigated BM entities (mean CD74 H-Score values: carcinoma NOS 79.4 (*n* = 8); colon carcinoma 51.4 (*n* = 10); breast carcinoma 42.5 (*n* = 31); RCC 133.9 (*n* = 18); NSCLC 73.2 (*n* = 56); SCLC 16.3 (*n* = 8); melanoma 26.5 (*n* = 96); others 71.1 (*n* = 9)). **d** Kaplan-Meier Survival analyses in the total BM cohort and in the subcohorts of NSCLC and melanoma after median split (median H-Score 20) according to CD74 expression

In a first step, we assessed protein expression profiles of CD74 in BM from distinct primary tumor entities. CD74 was expressed by tumor cells (Fig. 1a, b) and by myeloid cells such as tumor associated microglia and macrophages (TAMs) as previously shown by our group in primary brain tumors [57]. We solely analyzed CD74 expression in tumor cells not in TAMs. CD74 tumor cell expression was heterogeneous among all investigated entities (Fig. 1a-c) with highest expression levels in BM of RCC and NSCLC (mean H-Score of 133.9 and 73.2) respectively. We detected lowest levels in SCLC (mean H-Score of 16.2). Despite the strong variation of mean values, we found high and low expressors in all investigated entities (Fig. 1c, median H-Score 20). Most interestingly, high CD74 expression on tumor cells was associated with prolonged patient overall survival after BM surgery in the total cohort as well as in our largest subcohorts of BM deriving from NSCLC and melanoma (Fig. 1d). Interestingly, CD74 expression in tumor cells was not associated with clinical parameters such as Karnofsky Performance Status (KPS) or in case of melanoma the Graded Prognostic Assessment (GPA) Score (Additional file 1: Figure S1). Furthermore, non-cerebral, peripheral melanoma metastases (skin metastases) did not show a significant survival benefit (Additional file 2: Figure S2a). In peripheral melanoma skin metastases, we did not find a significant difference in CD74 expression between tumor stage III and tumor stage IV patients (Additional file 2: Figure S2b). Furthermore, we had the opportunity to analyze primary tumors (*n* = 10), peripheral metastases (*n* = 4) as well as BM (*n* = 56) of NSCLC patients. We did not observe any significant differences between these different stages of disease with regard to CD74 expression (Additional file 2: Figure S2c). Matched-pair analysis of CD74 expression in primary tumors and BM did not reveal a clear trend (Additional file 2: Figure S2d).

### CD74 expression in BM is associated with immune cell infiltration

**Fig. 2:**
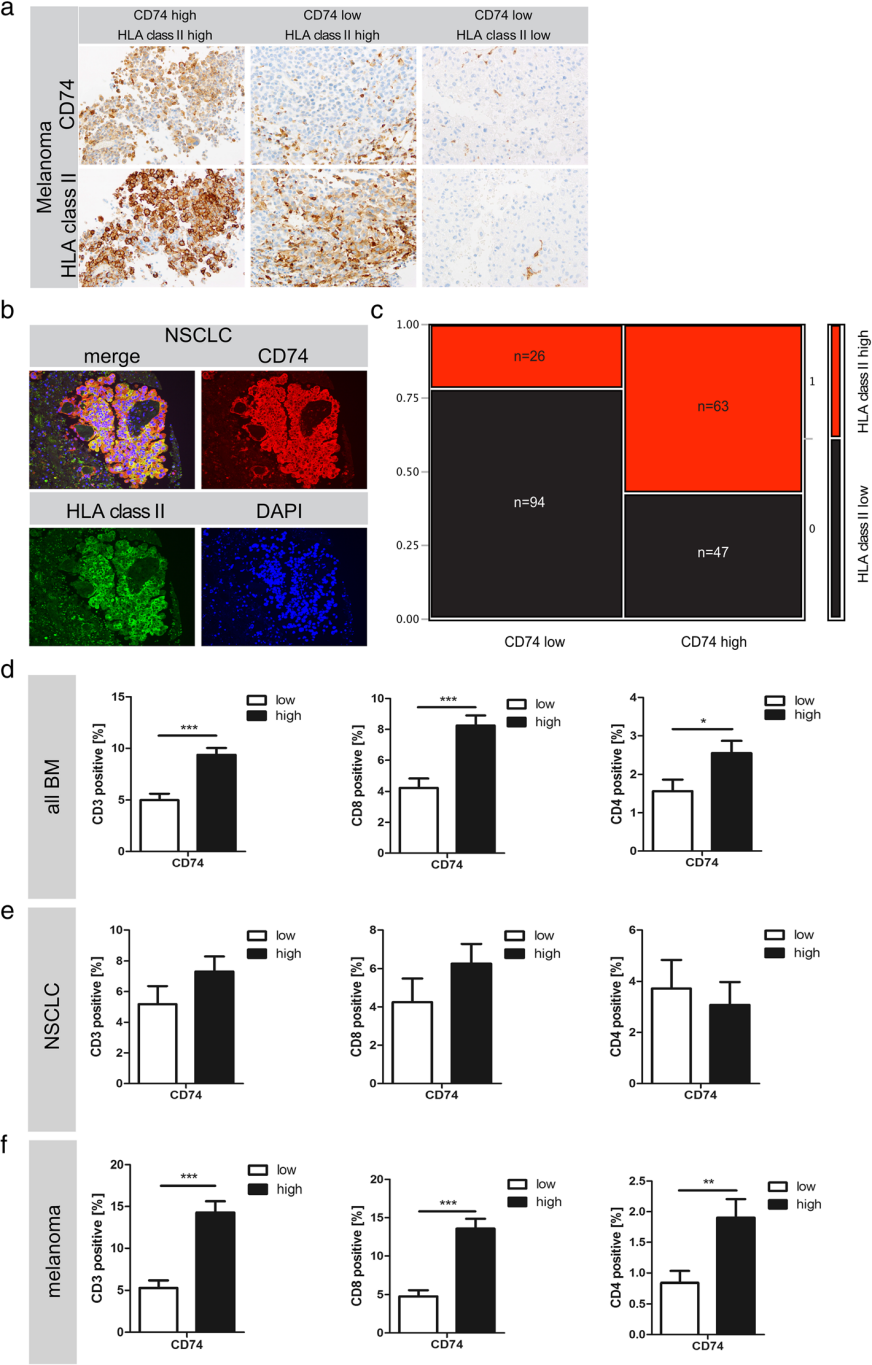
CD74 association with HLA class II and TILs. **a** Immunohistochemical staining against HLA class II members and CD74 revealing a heterogeneous expression pattern in tumor cells of BM from melanoma. CD74 positive myeloid cells were excluded from further analysis. **b** Immunofluorescent doublestaining indicating an overlap of CD74 and HLA class II in the majority of tumors. **c** Contingency table showing, that more than 50% of CD74 high tumors also showed high levels of HLA class II. **d**, **e** and (**f**) illustrating the association between CD74 expression and TILs in different BM entities

As CD74 is a HLA class II chaperone molecule which guides and stabilizes assembly as well as influences the intracellular transport of HLA class II molecules, we next investigated whether CD74 expression is associated with HLA class II expression profiles. Despite heterogeneous expression patterns of CD74 and HLA class II in individual cases (Fig. 2a, b), H-Score values strongly correlated between CD74 and HLA class II in our total cohort of BM (Spearman’s ρ 0.5440, *p* < 0.0001; Fig. 2c). More than half of the investigated BM showing high CD74 expression also showed high HLA class II values (Fig. 2c). However, CD74 low expressors did not necessarily show low expression levels for HLA class II expression. Interestingly, HLA class II expression alone showed no significant association with overall survival (Additional file 3: Figure S3a). Moreover, HLA class II expression was significantly reduced in BM as compared to their primary tumors (*p* < 0.002, Additional file 3: Figure S3b), which might indicate that the mere amount of HLA class II molecules is not the crucial step for a functional immune response in BM.

We recently found that the mere amount of TILs (CD3-positive and CD8-positive lymphocytes as well as subsets of these cells) is not associated with patient survival [16]. In the total cohort of BM as well as in the subcohort of melanoma BM the positive prognostic marker CD74 on tumor cells is nevertheless significantly associated with increased numbers of TILs (including CD3-, CD4- and CD8-positive TILs) (Fig. 2d, e). In the cohort of NSCLC BM, CD74^high^ expressors also showed a trend towards increased CD3- and CD8-positive TILs, while we did not observe significant differences with regard to CD4-positive cells.

Current therapeutic strategies focus on targeting the immune checkpoint axis PD-1 and PD-L1. Although the expression of these molecules is not prognostic for BM [16] and e.g. resected NSCLC [49], we investigated the association of PD-1/CD8 positive TILs as well as PD-L1 positive tumor cells with CD74 in BM. We only found a weak significant positive correlation between PD-L1 and CD74 in BM from melanoma (Table 1).

**Table 1:** Association between CD74 expression, PD-L1 expression and PD1/CD8-positive TILs in BM

	PD1/CD8 all	PD1/CD8 NSCLC	PD1/CD8 melanoma	PD-L1 all	PD-L1 NSCLC	PD-L1 melanoma
CD74 all	ρ=0.0251 *p* = 0.7184			ρ=0.0900 *p* = 0.1815		
CD74 NSCLC		ρ=0.0190 *p* = 0.8980			ρ=0.0793 *p* = 0.5613	
CD74 melanoma			ρ=0.3947 *p* = 0.5108			ρ=0.2912 *p* = 0.0072

Correlation analyses (Spearmen’s ρ and corresponding *p*-values) between CD74 expression, PD-L1 and PD1/CD8-positive TILs in the total cohort of BM, melanoma BM and BM from NSCLC

### CD74^high^ and TIL^high^ tumors show a differential methylation pattern in which genes associated with antigen processing and presentation are enriched among differentially methylated CpGs

**Fig. 3:**
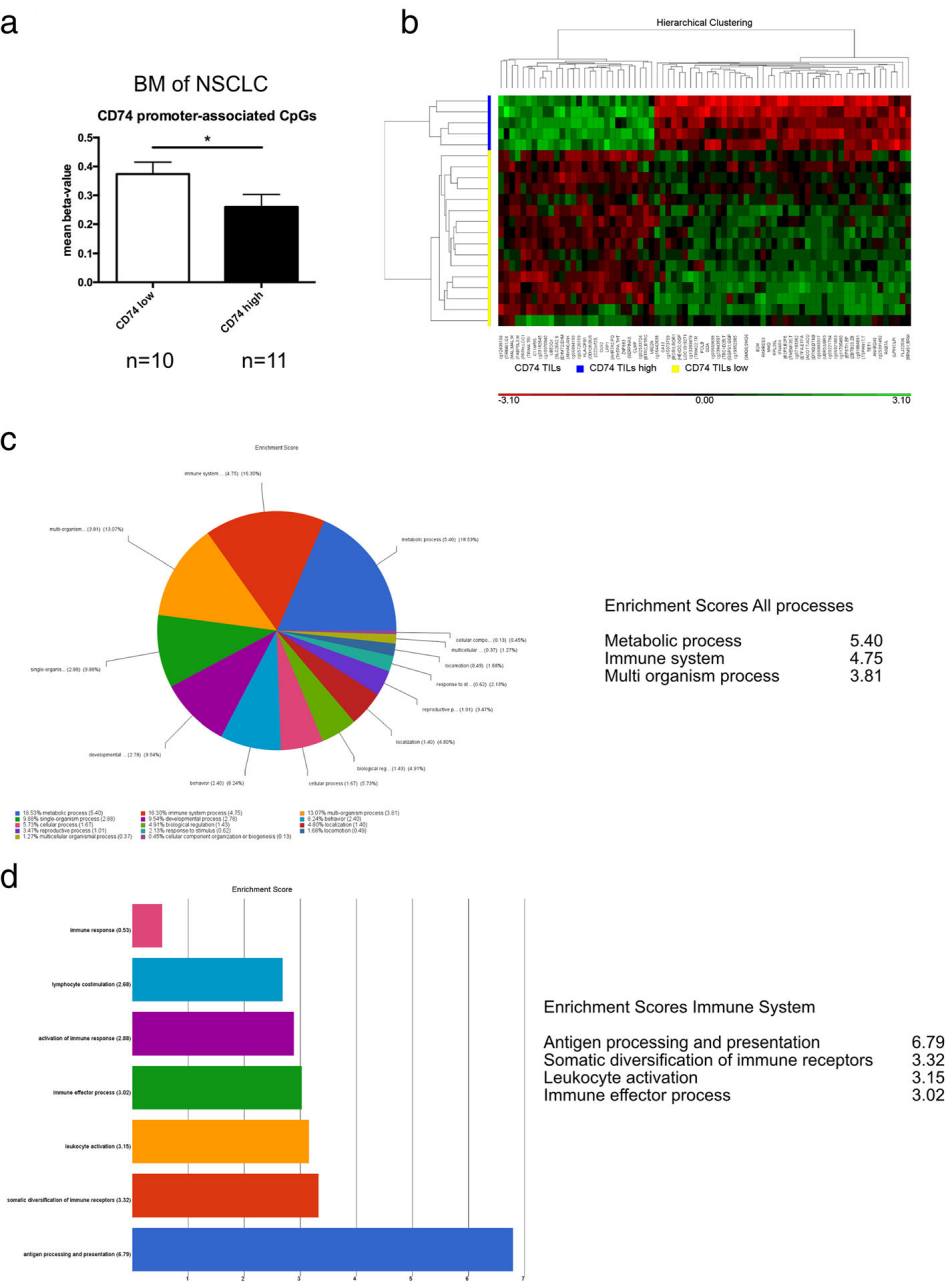
CD74 promoter methylation and whole DNA methylation patterns in NSCLC BM. **a** Mean beta-values of promoter-associated CpGs in 21 BM from NSCLC. CD74 low expressors are associated with significantly increased mean beta-values. **b** Differentially methylated CpGs of 21 BM from NSCLC, stratified by the combinatory parameters CD74 high + TILs high (CD74 TILs high, *n* = 5, blue) versus tumors not showing these combined features (CD74 TILs low, *n* = 16, yellow). Hierarchical cluster analysis showing 74 differentially methylated CpGs (M-values are shown, unadjusted *p*-value < 0.0001, Additional file 4: Table S1). **c** Gene ontology enrichment analysis of biological processes, (**d**) gene ontology enrichment analysis of Immune System processes

To further assess heterogeneous CD74 expression patterns among the same tumor entity we investigated a potential epigenetic regulation of CD74 expression in BM. We investigated promoter-associated CpGs for hypo- or hypermethylation in 21 NSCLC BM. As expected, mean beta-values were rather low, as CD74 is strongly expressed by TAMs as previously shown [49]. Nevertheless, we detected significantly reduced beta-values in CD74^high^ versus CD74^low^ protein expressors (Fig. 3a), indicating a potential epigenetic regulation of CD74 expression in tumor cells from BM. We further asked whether there might be a differential methylation pattern between BM with a potential highly functional immune response, which we hypothetically defined as CD74^high^ and TIL^high^ tumors (CD74 TIL high) and tumors not showing both of these features (CD74 TIL low). Differential methylation analyses revealed 74 CpG sites being differentially methylated between both groups (Fig. 3b). Gene ontology enrichment analysis of differentially methylated CpGs of NSCLC BM showed a strong enrichment for processes of immune response and especially for antigen processing and presentation (Fig. 3c, d) (differentially methylated CpG sites are shown in Additional file 4: Table S1).

### CD74 is expressed in metastatic cell lines of different entities without showing a relevant surface expression

**Fig. 4:**
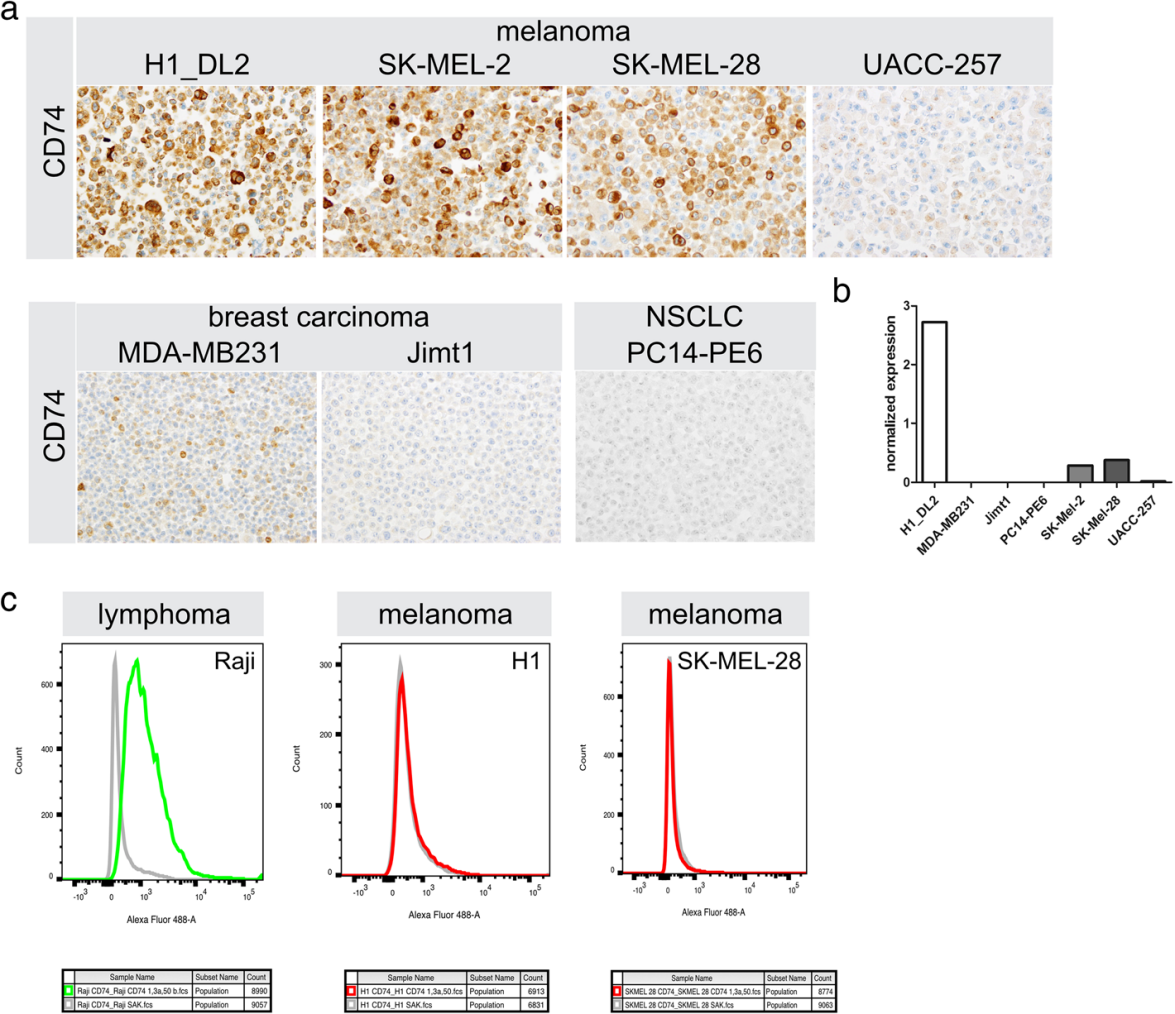
CD74 expression in vitro using brain seeking BM cell lines. **a** Immunocytochemistry against CD74 in different brain seeking human BM cell lines. **b** Normalized results of CD74 transcript expression using qPCR. H1 and H1_DL2 cell line showed similar results, both on protein and transcript level (data not shown). **c** FACS analyses of unfixed cell lines. Positive control cell line Raji showing CD74 expression on the cell surface, while H1 and SK-MEL-28 cell lines don’t show CD74 on the cell surface

We assessed CD74 expression levels in tumor cell lines from different entities (H1 and H1_DL2 (brain seeking cell line from melanoma BM), SKMEL-2 (skin metastatic melanoma), SKMEL-28, UACC-257 (both melanoma), MDA-MB-231, Jimt1 (both brain seeking clones of pleural metastatic breast cancer), PC-14 (brain seeking clone of lung adenocarcinoma)). We were able to detect CD74 expression by immunocytochemistry notably in H1_DL2, SKMEL-2 and SKMEL-28, mildly in UACC-257 and MDA-MB-231 but not significantly present in Jimt1 and PC-14 (Fig. 4a) cells. H1 cells yielded similar results as H1_DL2 (data not shown). qRT-PCR showed highest RNA levels for H1_DL2 melanoma cells (Fig. 4b). Besides its role as an assembly and stabilization molecule during HLA class II processing, CD74 has also been described as a MIF receptor, acting on the cell membrane [26]. To further analyze whether CD74 was expressed on the cell membrane we performed FACS analysis of brain seeking human BM cell lines. Interestingly, we did not detect CD74 cell surface expression in unfixed H1 melanoma cells indicating a functional restriction to mainly intracellular compartments (Fig. 4c).

### CD74 knock-down may influence HLA class II-mediated antigen presentation by reducing the complexity of the HLA class II peptidome

CD74 expression appeared to be restricted to intracellular compartments of H1 brain seeking melanoma cells. CD74 expression is further associated with the presence of TILs. We thus hypothesized that CD74 may contribute to antigen processing and presentation in BM. We hence asked whether an siRNA-mediated knockdown of CD74 would affect the HLA class II processing machinery.

**Fig. 5:**
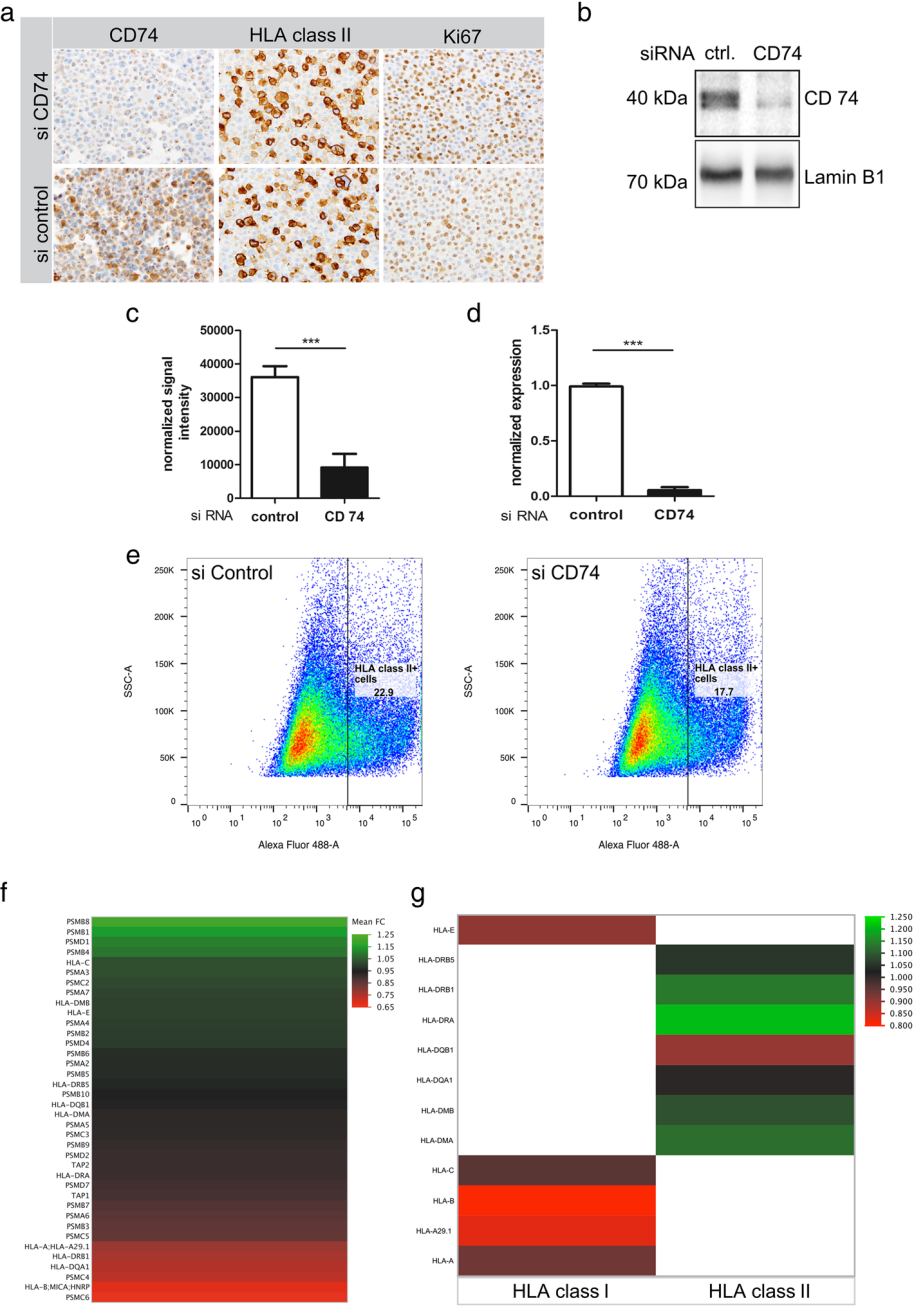
The influence of CD74 siRNA mediated knockdown on HLA class I and II machinery in H1 brain metastatic melanoma cells. **a** Immunocytochemistry of H1 melanoma cells against CD74, HLA class II molecules and Ki67. **b** + **c** Western blot analysis of siRNA-mediated CD74 knock-down versus siRNA-control with (**c**) protein quantification normalized against Lamin B1. **d** qRT-PCR analysis of siRNA-mediated CD74 knock-down versus control. **e** FACS analyses against HLA class II molecules on H1 melanoma cells. Differential transcript expression of H1 siRNA CD74 versus control siRNA (**f**, **g**). **f** TaqMan® array focusing on the transcriptome of the human antigen processing and presentation machinery by HLAs, (**g**) excerpt of micro-array data with regard to HLA class I and II transcripts

CD74 siRNA knockdown in H1 brain metastatic cells (Fig. 5a-d) did not influence HLA class II expression on protein level (Fig. 5a), transcriptional level (as assessed by TaqMan® array designed for the detection of the human antigen processing and presentation machinery by HLAs (Fig. 5f) corroborated by RNA-microarray (Fig. 5g)) nor did it influence HLA class II expression on the tumor cell surface as assessed by FACS analysis (Fig. 5e). Interestingly, none of the crucial HLA class I and II processing factors showed a significant regulation upon CD74 knockdown on transcriptional level (Fig. 5f).

**Fig. 6:**
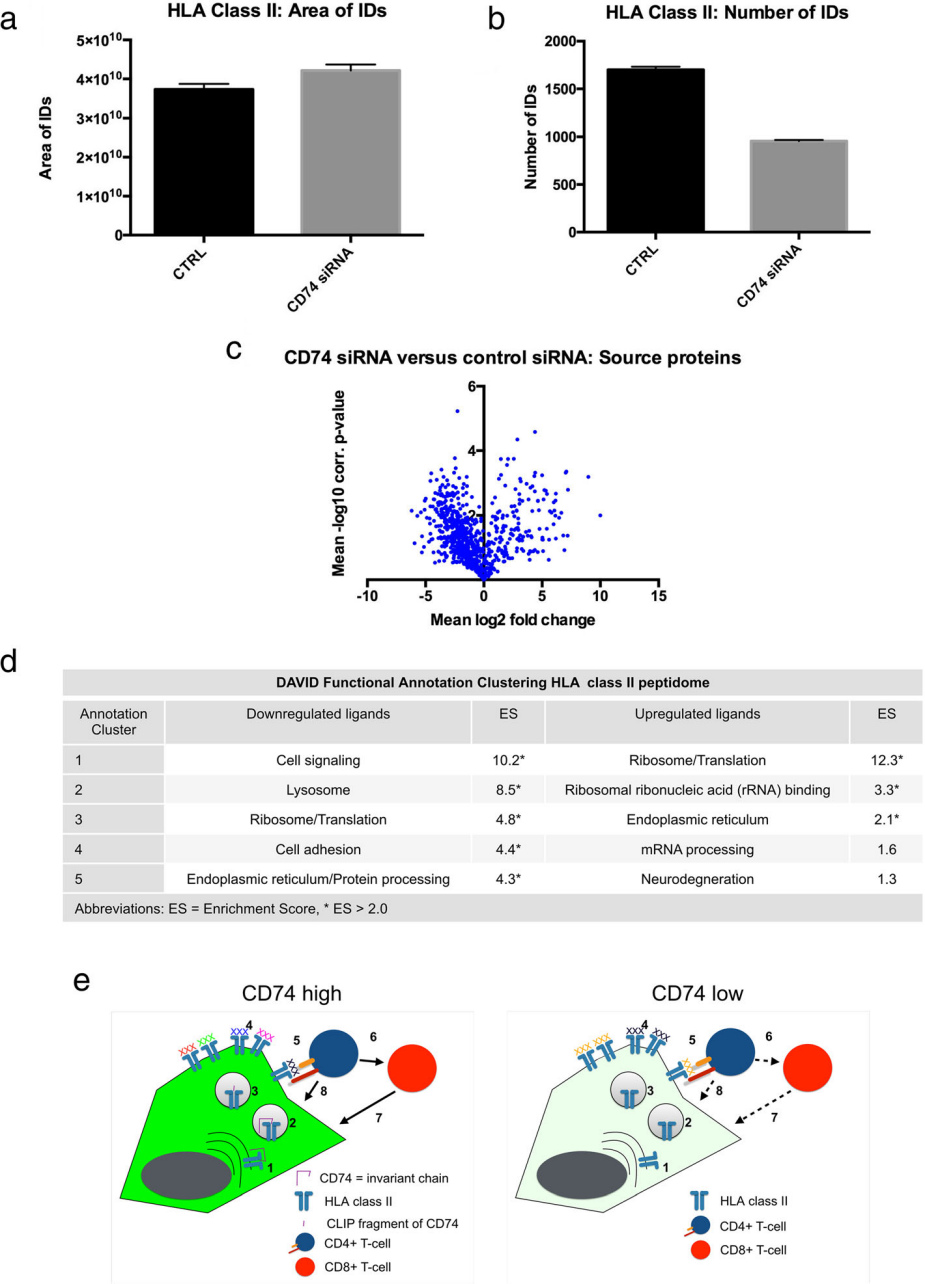
Effects of siRNA mediated CD74 knockdown on the HLA class II peptidome of H1 brain metastatic melanoma cells. **a** Summed MS1 intensities and (**b**) Number of identifications of HLA class II ligands in label-free quantitation mass spectrometry (error bars represent standard error of the mean (SEM). **c** Volcano plot of differentially presented source proteins in CD74 knockdown vs control (**d**) DAVID Functional Annotation Clustering of differentially represented HLA class II source proteins. **e** Schematic illustration of CD74 functions (left) and consequences of CD74 knockdown/downregulation (right) in brain metastatic tumor cells. 1: HLA class II and invariant chain complex in the endoplasmic reticulum and Golgi apparatus, 2: HLA class II compartment, 3: Processing of invariant chain by proteases, CLIP fragment remains and is exchanged for an antigenic peptide, 4: Complex antigens are expressed on the tumor cell surface when CD74 is highly expressed, 5: Tumor cell - CD4-positive lymphocyte interaction, 6: Recruitment of CD8-positive T-cells, 7: direct lysis by CD8 or 8: CD4-positive cells. Dotted lines illustrate impaired tumor cell – lymphocyte interactions. XXX denote HLA class II ligands. Multiple colors of ligands denote high peptidome complexity

As CD74 knockdown did neither alter the amount of HLA class II molecules nor affect central regulators of the HLA class I and II family on transcriptional level, we assessed whether a CD74 knockdown directly affects antigen presentation by altering the HLA class II peptidome composition. Label-free quantitation mass spectrometry of the HLA peptidome of H1 brain metastatic tumor cells suggests that the overall amount of class II peptides - approximated by the summed signal intensity of all peptide identifications – does not substantially differ between control and CD74 knockdown condition (Fig. 6a). The number of unique class II peptide identifications on the other hand was reduced by 47% in CD74 siRNA treated H1 cells compared to control indicating a reduced complexity of the class II peptidome (Fig. 6b), whereas HLA class I peptidome composition was not affected (data not shown). Volcano plot analysis of differential source protein presentation in the class II peptidome (Fig. 6c) revealed 52/781 (6.7%) source proteins to be significantly overrepresented (≥2 average fold-change in LFQ signal intensity of corresponding class II peptides, avg. *p*-value≤0.01) on CD74 siRNA treated H1 cells, whereas 101/781 (12.9%) showed down-modulation. Functional Annotation Clustering revealed molecules of cell signaling pathways, of the lysosomal and of the ribosomal compartment as main downregulated HLA class II ligands upon CD74 knockdown (Fig. 6d). Interestingly, most upregulated ligands upon CD74 knockdown were found in the group of ribosomal proteins as well as rRNA binding molecules, where to date relatively few neoantigens have been found [14].

## Discussion and conclusions

BM are the most frequent CNS tumors and constitute a fatal disease, which is often accompanied with a short disease interval and high patients’ disability. Still, therapeutic strategies are limited and include whole brain radiotherapy (WBRT), stereotactic irradiation (SI), surgery, chemotherapy (CTX) or a combination of different approaches.

In recent years immunomodulatory therapeutic strategies mainly targeting immune checkpoints led to a considerable improvement of patient survival and quality of life and have therefore been rapidly approved for therapy in different cancer entities [38]. These drugs re-invigorate an exhausted lymphocytic immune response against the tumor cells. Thus, a successful response strongly depends on the cellular composition of the microenvironment. In peripheral tumors the composition of lymphocytic infiltration is a well accepted prognostic marker and in case of breast cancer TILs have already reached the status of an established biomarker [17]. In CNS tumors and especially BM the relevance of the mere amount of TILs is however still controversial and might not be a sufficient prognostic tool [5, 16].

In the present study, we showed that CD74 expression in tumor cells, a major assembly and stabilization molecule of the HLA class II machinery, is a strong prognostic factor for BM patients. In contrast to peripheral metastases we observed this prognostic value in the cohort of all investigated BM and in the subcohorts of NSCLC and melanoma BM. This indicates a specific role for CD74 functionality in the microenvironment of BM. Interestingly, CD74 expression has been described to be prognostic in particular subcohorts of peripheral tumors such as basal like and triple negative breast cancer, but not in the total investigated cohort in these studies [11, 54] indicating, that there might be a more global genetic or epigenetic regulatory event. In line with the heterogeneous CD74 expression levels among the same tumor entity, we found CD74 promoter methylation to be associated with low CD74 protein expression in NSCLC BM, indicating an epigenetic regulatory event in these tumors. Rather low apparent levels of promoter methylation in these tumors can be explained by CD74 expression not being restricted to tumor cells. Instead CD74 is a major member of the HLA class II machinery which is highly expressed by antigen presenting cells such as TAMs [57]. A total CD74 promoter methylation in tumor tissue is therefore most unlikely. Interestingly, CD74 and HLA class II expression have been shown to be reactivated upon treatment of ovarian cancer cells with histone deacetylase and DNA methyltransferase inhibitors, which in turn lead to a reduced tumor growth in an experimental in vivo model [50]. The underlying mechanisms of this favorable reactivation of HLA class II members as well as downstream effects still remain unclear. Epigenetic regulation of the HLA class II machinery by class II transactivator protein (CIITA) has been described in various cancer cell lines. Methylation of CIITA promoter IV seems to reduce interferone-gamma-inducible HLA-DR expression on cancer cells [36, 44, 56]. Moreover, about 3% of the HLA ligandome was recently found to be detectable both on HLA class I and II. Processing of such peptides detected on both class I and class II was proposed to require the cellular class II presentation machinery [32], implicating that a loss of CD74 expression could also affect presentation of neoantigens on HLA class I.

In our current study, we found CD74 tumor cell expression to be associated with the frequency of TILs. Nevertheless the mere amount of TILs and subsets (including PD-1-positive TILs) has not been prognostic for BM patients in one of our previous studies [16]. Unlike CD74 expression, the mere HLA class II expression was also not prognostic in our investigated BM cohorts (Additional file 2: Figure S2), although (similar to CD74) HLA class II expression is considered as a potential prognostic factor for some peripheral cancer entities like ovarian cancer or triple negative and basal like breast cancer in the aforementioned studies [11, 54]. Interestingly, we found a significantly reduced expression of HLA class II molecules in BM as compared to their primary tumors, raising the question whether the mere amount of HLA class II molecules on tumor cells might be limiting for a functional immune response in the microenvironment of BM. However, immune responses do not only depend on the available antigen-presenting molecules. Instead, the quality or complexity of the presented HLA peptidome is also crucial for an adequate immune response.

Our results suggest, that upon CD74 knockdown in a brain metastatic melanoma cell line the complexity of the HLA class II peptidome was strongly reduced, while the mere amount of HLA class II molecules did not appear to vary between knockdown and control condition, nor did we observe significant changes of HLA class I and II transcripts upon CD74 knockdown. This suggests that CD74 directly influences the diversity of the HLA class II peptidome on tumor cells. The prognostic significance of CD74 hence suggests that the complexity of the HLA class II peptidome might be critical for a functional HLA class II dependent immune response in BM. However, while the search for tumor-specific antigens has prompted thorough investigations of the HLA class I peptidome, the HLA class II peptidome of cancer cells has only been explored in much lesser detail, which may be due to the old and grossly oversimplified concept that endogenous antigens are presented on HLA class I while HLA class II would mainly present exogenous antigens. Nevertheless, previous studies already identified HLA class II dependent cancer-specific phosphopeptides which could become targets for immunotherapy. Likewise, a recent study identified cancer specific antigens on HLA class I and II molecules even in low mutated tumors such as ovarian carcinomas [10, 39]. In fact, only about 20% of antigens presented on HLA class II seem to derive from exogenous sources incorporated by endocytosis, while the remainder consists of endogenous proteins from plasma, lysosomal or endosomal membraneous compartments, and to a minor extent also from the cytoplasm, nucleus and golgi apparatus [42]. Interestingly, upon CD74 knockdown in melanoma brain metastatic cells we observed a significantly decreased presentation of HLA class II source proteins belonging to cell signaling pathways and the lysosomal compartment, while ribosomal sources were upregulated. Whether these downregulated peptides are the crucial peptides responsible for an anti-tumor response in CD74 high expressing tumors remains an open question which should be addressed in future studies.

Since different cancer entities show different biological behavior especially with regard to CD74 expression and its function, there is no universal answer whether CD74 is mainly associated with aggressiveness or positive prognosis. Here we could demonstrate that BM with high CD74 expression show a better clinical course. Although we investigated a fair number of samples (especially in our largest subgroup of melanoma BM patients), our patient cohorts are still limited especially regarding subgroups of other cancer entities. Thus, results of survival analyses of subgroups should be confirmed in larger subcohorts of future studies. Furthermore, we show that this expression might be epigenetically regulated via CD74 promoter methylation at least in BM deriving from NSCLC. Additional studies in other BM entities would be reasonable and a potential association of a differential whole DNA methylation pattern with a functional immune response should be target of future studies. On a functional level we showed that CD74 regulates tumor cell HLA class II immune peptidome complexity, which may be a central immunological event for a clinically relevant HLA class II restricted anti-tumor response in BM. To answer this question properly, the exact role of CD74 in tumor cells for functional immune responses including the impact on the function of different effector cell subsets like TILs and NK-cells has to be addressed in detail in additional studies. HLA class II peptidome analyses in patient samples of CD74^high^ versus CD74^low^ expressors are likewise highly relevant and unfortunately a technical limitation of the current study. Moreover, given that recent studies point out the importance of an unimpaired antigen-processing machinery for a successful immunotherapy response [35] CD74 in cancer cells should also be explored as a putative predictive marker for immunotherapies.

## Additional files

Additional file 1: **Figure S1.** Clinical data of the different BM cohorts and association of CD74 expression with clinical parameters. Correlation analyses were performed using Spearmen’s correlation analyses (Spearmen’s ρ and corresponding *p*-values are depicted). (JPEG 1333 kb)

Additional file 2: **Figure S2.** CD74 expression in different stages of melanoma (a, b) and NSCLC (c). Matched-pairs analysis of primary tumors and BM (different primary tumor entities) (d). (JPEG 1243 kb)

Additional file 3: **Figure S3.** (a) HLA class II dependent Kaplan-Meier survival analyses in the total BM cohort as well as in the two largest subcohorts of NSCLC and melanoma. (b) Matched-pairs analysis of primary tumors and BM (different primary tumor entities). (JPEG 2138 kb)

Additional file 4: **Table S1.** Table showing differentially methylated CpGs between BM of NSCLC with a highly functional immune response, defined as CD74^high^ and TIL^high^ tumors (CD74 TIL high) and tumors not showing both of these features (CD74 TIL low). (CSV 1 kb)
