# Supplementary figures and images for: CD74 regulates complexity of tumor cell HLA class II peptidome in brain metastasis and is a positive prognostic marker for patient survival
Source: Acta Neuropathol Commun. 2018 Mar 1;6:18. doi: 10.1186/s40478-018-0521-5 (PMC5831742; doi:10.1186/s40478-018-0521-5)

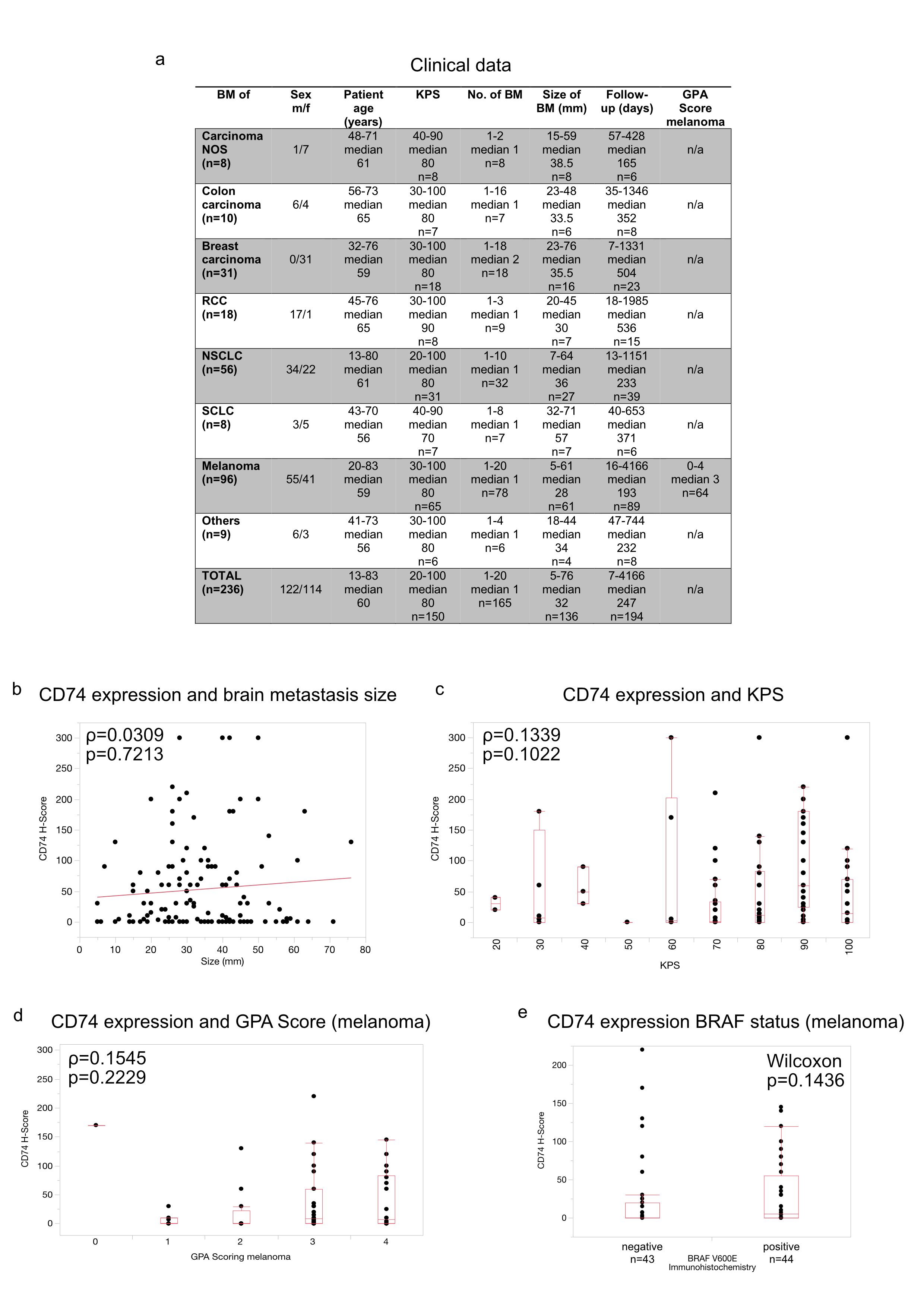

Supplement: Supplementary file 1 — Figure S1. Clinical data of the different BM cohorts and association of CD74 expression with clinical parameters. Correlation analyses were performed using Spearmen’s correlation analyses (Spearmen’s ρ and corresponding p-values are depicted). (JPEG 1333 kb) [file 40478_2018_521_MOESM1_ESM.jpg]

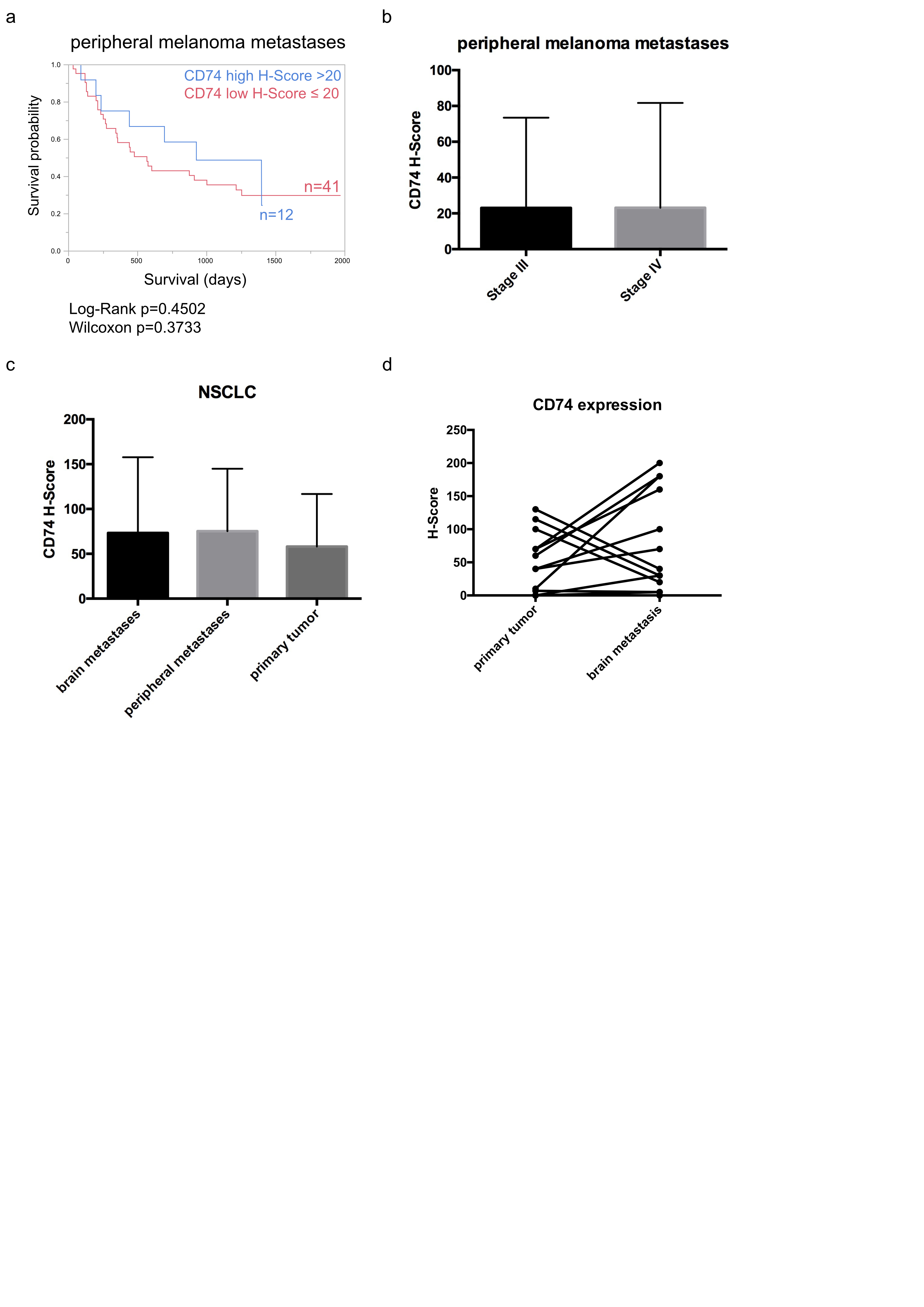

Supplement: Supplementary file 2 — Figure S2. CD74 expression in different stages of melanoma (a, b) and NSCLC (c). Matched-pairs analysis of primary tumors and BM (different primary tumor entities) (d). (JPEG 1243 kb) [file 40478_2018_521_MOESM2_ESM.jpg]

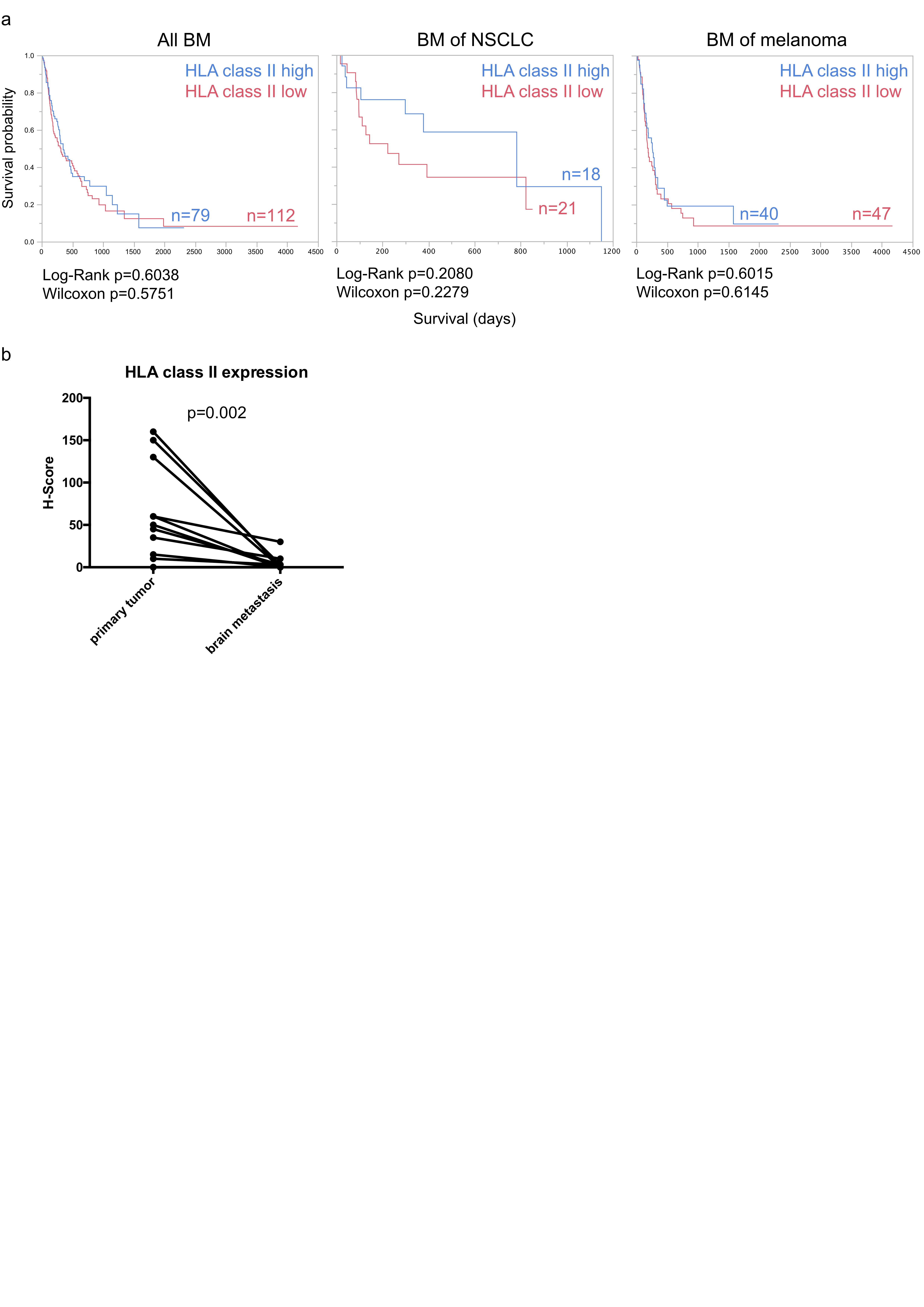

Supplement: Supplementary file 3 — Figure S3. (a) HLA class II dependent Kaplan-Meier survival analyses in the total BM cohort as well as in the two largest subcohorts of NSCLC and melanoma. (b) Matched-pairs analysis of primary tumors and BM (different primary tumor entities). (JPEG 2138 kb) [file 40478_2018_521_MOESM3_ESM.jpg]
